# SHORT-TERM FOETAL IMMOBILITY TEMPORALLY AND PROGRESSIVELY AFFECTS CHICK SPINAL CURVATURE AND ANATOMY AND RIB DEVELOPMENT

**DOI:** 10.22203/eCM.v037a03

**Published:** 2019-01-15

**Authors:** A. Levillain, R.A. Rolfe, Y. Huang, J.C. Iatridis, N.C. Nowlan

**Affiliations:** 1 Department of Bioengineering, Imperial College London, London, UK; 2 Department of Zoology, Trinity College Dublin, Dublin, Ireland; 3 Department of Orthopaedics, Icahn School of Medicine at Mount Sinai, New York, NY, USA

**Keywords:** Congenital spine deformities, congenital scoliosis, embryo, paralysis, foetal movement, biomechanics, vertebrae

## Abstract

Congenital spine deformities may be influenced by movements *in utero*, but the effects of foetal immobility on spine and rib development remain unclear. The purpose of the present study was to determine (1) critical time-periods when rigid paralysis caused the most severe disruption in spine and rib development and (2) how the effects of an early, short-term immobilisation were propagated to the different features of spine and rib development. Chick embryos were immobilised once per single embryonic day (E) between E3 and E6 and harvested at E9. To assess the ontogenetic effects following single-day immobilisation, other embryos were immobilised at E4 and harvested daily between E5 and E9. Spinal curvature, vertebral shape and segmentation and rib development were analysed by optical projection tomography and histology. The results demonstrated that periods critical for movement varied for different aspects of spine and rib development. Single-day immobilisation at E3 or E4 resulted in the most pronounced spinal curvature abnormalities, multiple wedged vertebrae and segmentation defects, while single-day immobilisation at E5 led to the most severe rib abnormalities. Assessment of ontogenetic effects following single-day immobilisation at E4 revealed that vertebral segmentation defects were subsequent to earlier vertebral body shape and spinal curvature abnormalities, while rib formation (although delayed) was independent from thoracic vertebral shape or curvature changes.

A day-long immobilisation in chicks severely affected spine and rib development, highlighting the importance of abnormal foetal movements at specific time-points and motivating targeted prenatal monitoring for early diagnosis of congenital scoliosis.

## Introduction

Congenital scoliosis (CS) is a condition of the postnatal spine occurring in 0.5-1 ‰ live births and is characterised by a lateral curvature of the spine (Giampietro et al., 2013). The occurrence of abnormal vertebral segmentation at birth is more common than CS and places the infant at risk of developing scoliosis later in life (Brand, 2008). Congenital malformations of the spine also include lordosis and kyphosis, which are defined as excessive inward (lordosis) or backward (kyphosis) curvatures of the spine in the sagittal plane (Lonstein, 1999). These malformations are often associated with rib anomalies (Ghandhari et al., 2015) and can lead to respiratory insufficiency, pulmonary and cardiac hypertension and spinal cord compression (Brand, 2008; Weston et al., 2006). Correction of such malformations is often necessary to prevent or reduce these associated risks, but these curvature defects can be rigid and resistant to correction as the spine grows (Brand, 2008). Early diagnosis of CS and identification of patients susceptible to scoliosis later in life would enable correction of spinal asymmetry before significant growth occurs. Although the aetiology of CS remains unclear, foetal movements play a key role in spine development (Kalampokas et al., 2012). For example, arthrogryposis [multiple abnormal joint contractures (Hall, 2014)], which is associated with decreased foetal movements, features abnormal spinal development in up to 31 % of patients (Yingsakmongkol and Kumar, 2000). A better understanding of the involvement of foetal movements in spine and rib development could provide earlier diagnosis of CS and improve management of the pathology.

Foetal immobility can have severe effects on the development of the musculoskeletal system. Foetal immobility can be caused by neurogenic or myopathic disorders, reduction of amniotic fluid or abnormal foetal position (Kowalczyk and Feluś, 2016; Nowlan, 2015). Numerous studies on the effects of abnormal foetal movements on limb development use pharmacologically paralysed chick embryos or genetically modified mammalian models (as reviewed by Nowlan, 2015). These studies identify abnormal ossification patterns, loss of tissue definition in joint regions and altered rudiment shape (Kahn et al., 2009; Nowlan et al., 2010; Roddy et al., 2011). Studies on the influence of foetal movements on spine and rib development are fewer, but reveal abnormal spinal curvature (Rolfe et al., 2017), vertebral fusion (Hosseini and Hogg, 1991; Murray and Drachman, 1969; Rolfe et al., 2017) and segmentation defects (Rolfe et al., 2017) following prolonged paralysis in chick embryos, as well as truncated ribs in mouse embryos with abnormal muscles (Braun et al., 1992; Henderson et al., 1999; Vivian et al., 2000). Rolfe et al. (2017), using a chick model, show that both the type of muscle forces and the timing of movements affect spine development. Prolonged rigid paralysis, where only dynamic forces are removed, severely disrupts spinal curvature, vertebral shape and segmentation, whilst flaccid paralysis, where both static and dynamic forces are removed, results in only subtle changes in vertebral shape (Rolfe et al., 2017). Moreover, prolonged rigid paralysis induced at or prior to embryonic day 5 (E5) results in more severe abnormalities in the spine than later rigid paralysis. Together, these studies highlight the importance of motion during spinal development and show that prolonged rigid paralysis at early gestational stages has the most severe effects on spinal formation. However, prolonged paralysis *in utero* is rare and it would be beneficial to identify critical timings of foetal movements which could affect spine and rib cage development and the dependencies among structures as they develop.

A short immobilisation can result in an abnormal development of the musculoskeletal system. Foetal movements start at approximately 7 gestational weeks in humans and include head and neck movements (as reviewed by Nowlan, 2015). Absence of foetal movements (foetal akinesia) lasting over 3 weeks may be sufficient to result in abnormal stretching of muscles and contractures of the associated joints (Kowalczyk and Feluś, 2016). In a case study of foetal akinesia deformation sequence, Witters et al. (2002) report distal arthrogryposis as early as 12 gestational weeks. However, in most studies on arthrogryposis, the absence of movements in the foetus are reported from scans performed only during the second or third trimester of pregnancy (as reviewed by Nowlan, 2015); in addition, guidelines addressing the question of when foetal movement should be monitored during pregnancy with the purpose of detecting foetal akinesia at critical time-points are lacking (Filges and Hall, 2013). There is no understanding of when foetal movements are most important for normal development of the human spine and, therefore, no opportunity to screen prenatally for foetuses at increased risk of CS and other developmental spinal abnormalities. Evidence exists of the co-dependence between certain aspects of spine and rib development (Ghandhari et al., 2015), as abnormal spine curvature, vertebral segmentation and rib formation are frequently cornorbid in both human conditions and animal models of foetal akinesia. However, there are no studies that investigate the dependence of different aspects of spine and rib development on each other in the context of abnormal foetal movements.

The aims of the present study were to vaiy the onset time of short-term foetal immobility and assessment timings of spinal curvature and vertebral and rib formation to determine (1) what were the critical time-periods for which rigid paralysis caused the most severe disruption in spine and rib development and (2) how the effects of an early, short-term rigid paralysis were propagated to the different features of spine and rib development. The hypothesis that the timings of the short-term immobilisation differentially affected various features of spine and rib development was tested by immobilising chick embryos for single days between E3 [when sclerotome cell migration is occurring (Shapiro, 1992)] and E6 [when vertebral segmentation is complete (Shapiro, 1992)]. The hypothesis that normal development of later aspects of spine development (such as rib formation) depended upon earlier developmental events was tested by following the effects of a single day of immobilisation for several days of subsequent development.

## Materials and Methods

### *In ovo* immobilisation

Fertilised eggs (Dekalb white, MedEggs, Norfolk, UK) were incubated at 37.5 °C in a humidified incubator. Experimental embryos were immobilised for 1 d with 100 μL of 0.5 % decamethoniumbromide (DMB; Sigma-Aldrich) in phosphate buffered saline (PBS) supplemented with 100 unit/mL antibiotic (penicillin-streptomycin; Sigma-Aldrich). DMB is a neuromuscular blocking agent that induces rigid paralysis, where contractions of all skeletal muscle fibres are sustained (Osborne et al., 2002). Two types of immobilisation regimen were administered (Table 1). To study the critical timings of foetal mobility, experimental embryos were immobilised once at E3, E4, E5 or E6 while controls were treated with 100 pL of PBS supplemented with 100 unit/mL antibiotic. All embryos were harvested at E9. To study the ontogenetic effects of single-day immobilisation at a critical time-point, experimental embryos were immobilised once at E4 (with controls being saline-treated on the same day) and, then, harvested daily between E5 and E9. As the two sets of experiments were performed at different times and as the external temperature and conditions can affect development of chick embryos (Hamburger and Hamilton, 1992), two sets of data for specimens treated at E4 and harvested at E9 were acquired. All experiments were performed in accordance with the European Legislation (Directive 2010/63/EU), according to which no license is required when working with embryos younger than ⅔ of gestation. Euthanasia and harvesting of each specimen were performed by cutting the vasculature surrounding the embryo and placing it in ice-cold PBS, followed by the careful dissection of the spine along with the associated dorsal ribs. As it was difficult to keep the ventral portion of the ribs intact due to its association with the proximal forelimb, this region was removed following dissection of the sternum and scapula. Therefore, only data from the dorsal portion of the ribs are presented.

### Skeletal preparation and three-dimensional (3D) imaging

Whole spines and ribs were stained with 0.015 % aldan blue in 95 % ethanol supplemented with 20 % glacial acetic add for 4-8 h and deared in 1 % KOH for 0.5-6 h. Images of the spedmens were taken both after staining and after clearing and specimens showing curvature change due to processing were exduded. However, curvature changes were observed only for few specimens at early stages (harvested at E5). Specimens were scanned using optical projection tomography (OPT) (Sharpe et al., 2002) and 3D surface representations were produced for each spine using ImageJ (Schneider et al., 2012).

### Sagittal spine curvature outlines

To visualise curvature changes in the spines, the 3D surface representations of each spine were rotated so that the vertebral bodies and spinous processes were visible and a line was traced along the centres of the vertebral bodies to obtain an outline trace of the sagittal plane curvature (Rolfe et al., 2017). Sets of outline traces were aligned at thoracic vertebra 1 (T1) and regions of pronounced kyphosis and/or lordosis, as compared to the age-matched control outlines, were identified. As a previous study reveals no significant changes in curvature in the coronal plane due to prolonged immobilisation (Rolfe et al., 2017), analysis focussed solely on lateral curvature.

### Quantitative analysis of curvature in the sagittal plane

The geometric curvature (GC), the inverse of the curvature radius (Vrtovec et al., 2008), was calculated for each vertebral body in the sagittal plane. The centre of each vertebra was identified from the 3D data following the method previously described by Rolfe et al. (2017). Then, a curve was fitted to the vertebral coordinates using a cubic smoothing spline function, which places a third-degree polynomial around each point to fit an accurate curve across the data-set (MathWorks®, R2015a, Natick, MA, USA). The GC is defined for an arbitrary position on the spine as the reciprocal to the radius *R* of the osculating circle in 3D at that position and represents the amount by which the 3D vertebral body line deviates from being straight. The GC was obtained as previously described (Vrtovec et al., 2008):

$$GC(p)=\frac{\left|\frac{dC(p)}{dp}\times \frac{d^{2}C(p)}{dp^{2}}\right|}{|\frac{dC(p)}{dp}{|}^{3}}=\frac{1}{R(p)},$$

where *C*(*p*) is the vector *[x(p), y(p)]*, giving the *x* and *y* coordinates of the curve as a function of the *p^th^* vertebra, *R(p)* is the radius of curvature and × denotes the vector cross-product.

### Spine height

Vertical spine length from cervical vertebra 8 (C8) to lumbar vertebra 7 (L7) was measured as a straight line from the centres of C8 and L7 vertebrae in the mid-sagittal section. C8 was chosen as the starting point as it was the first vertebra visible on all scans.

### Vertebral wedging

**Fig. 1.**
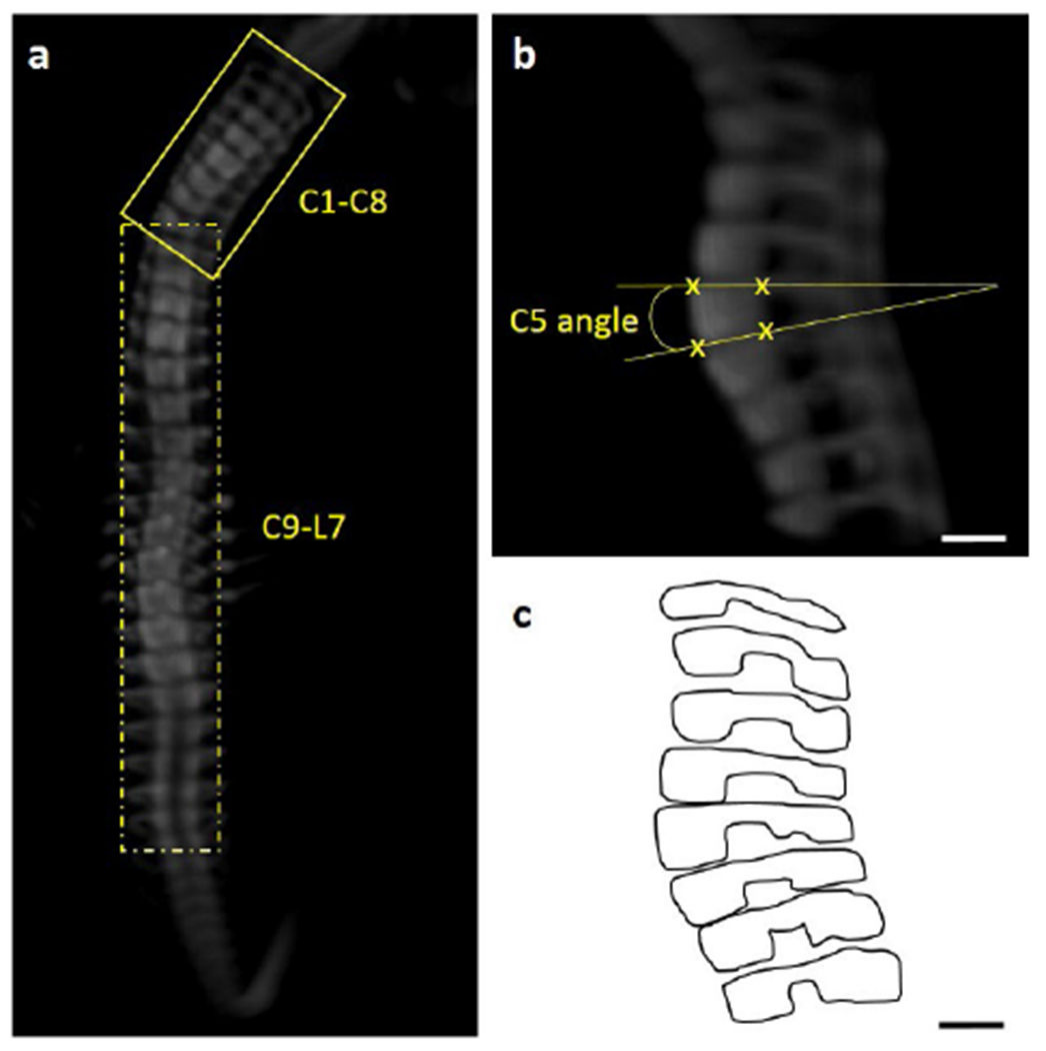
Illustration of methods used to measure the vertebral body angle. (**a**) Representative frontal 3D view of a control spine showing the two spinal segments (C1-C8 and C9-L7) that were cropped (yellow boxes) and aligned in the sagittal plane, (**b**) Sagittal 3D view of cervical spine segment (C1-C8) in (**a**). Yellow lines show how the vertebral body angle of cervical C5 was calculated. (**c**) Sagittal outlines of the vertebrae created from (**b**). Scale bars: 200 μm.

To measure vertebral wedging angles, spinal segments were first aligned in a local sagittal plane following a method similar to that described by Newell et al. (2017). Briefly, 3D representations were rotated in a frontal plane so that the anterior aspects of the vertebral bodies were in the front and the posterior and lateral portions were out of view. Then, spinal segments belonging to the same sagittal plane were cropped and rotated in their local sagittal plane (Fig. 1a). For each individual vertebra in the cervical (from cervical vertebra C8), thoracic and lumbar regions, wedging of vertebral bodies were quantified by measuring the angle created at the intersection of lines drawn along the superior and inferior endplate surfaces (Rolfe et al., 2017) (Fig. 1b) and sagittal outlines of vertebrae were produced (Fig. 1c). Due to normal variability in vertebral shape (Newell et al., 2017), vertebrae were considered to be wedged when the angle was superior to 10° and to be fused when the separation between two (or more) adjacent vertebrae could not be identified. Then, the numbers of wedged and fused vertebrae were determined for each specimen, along with the total number of abnormal vertebrae (wedged or fused vertebrae).

### Rib development

**Fig. 2.**
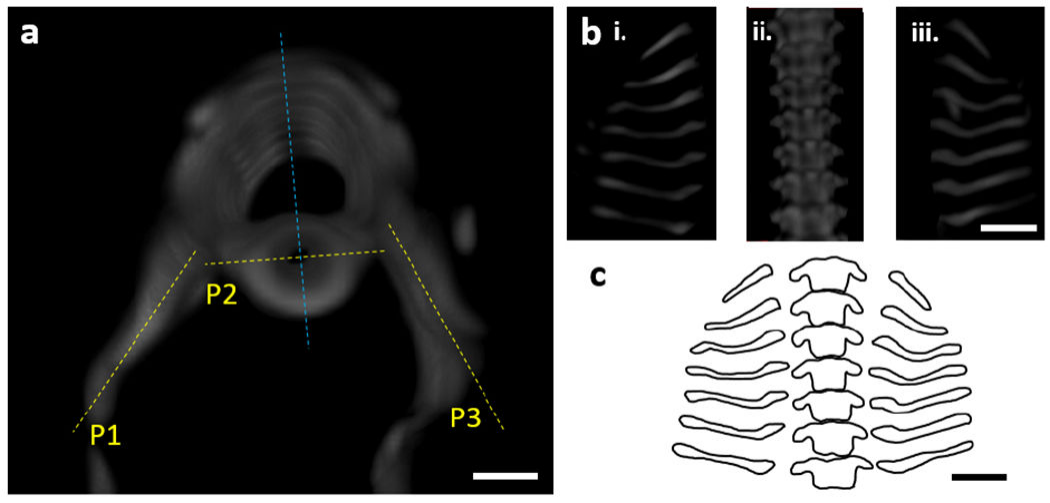
Illustration of the method used to create rib outlines. (**a**) Representative axial 3D view of thoracic spine segment (T1-T7) of a representative control specimen with associated dorsal ribs. Yellow lines show the different planes (P1, P2 and P3) in which vertebra and rib outlines were drawn. Line corresponding to P2 is perpendicular to the blue line running through the spinous processes and the centre of the notochord. (**b**) 3D view of thoracic spine segment and ribs in (**a**) planes P1 (i), P2 (ii) and P3 (iii). (**c**) Rib and vertebrae outlines created from (**b**). Scale bars: 500 μm.

To analyse individual ribs and associated thoracic vertebrae, the 3D surface representations of the thoracic region of each specimen with intact ribs were rotated in the axial plane (Fig. 2a) and visualised in three parts: (P1) the plane running through the length of the right rib; (P2) the frontal thoracic plane, which is perpendicular to the plane running through the spinous process and the centre of the notochord; (P3) the plane running through the length of the left ribs (Fig. 2b). Outlines of vertebral ribs (Aoyama et al., 2005) and thoracic vertebrae were produced from 3D representations for each individual specimen (Fig. 2c). The number of specimens with absent ribs or displaying rib fusion was calculated for each group. Additionally, for each specimen, curved length, *L_C′_*, and tortuosity [the property of a curve which is full of twists and turns (Bullitt et al., 2003)] of the fifth left vertebral rib were measured using Paraview (Kitware Inc., Clifton Park, NY, USA) (Ayachit, 2015) after segmentation in Mimics (Mimics 19.0, Materialise, Leuven, Belgium). The fifth left rib was chosen as this rib was present in all but one specimen. Tortuosity was evaluated using the inflection count metric (ICM), defined according to the following equation (Bullitt et al., 2003):

$$ICM=N\frac{L_{C}}{L_{S}},$$

where *N* is the number of inflection points and *L_s_* is the linear distance between the endpoints of the vertebral rib.

### Vertebral segmentation

Histological analysis of vertebral segmentation, the distinct spatial separation of cartilaginous vertebrae, was performed following either paraffin or optimal cutting temperature (OCT) embedding, sectioning (range 8-14 μm) and staining with 0.025 % alcian blue in 3 % acetic add (for cartilage) followed by 1 % picrosirius red (for collagen). Sections were imaged by transmitted illumination using a light microscope (Yenway EX30; Life Sdences Microscope, Glasgow, UK). For each specimen, the proportion of fused or partially segmented joints (vertebral bodies or spinous processes) in each region (cervical, thoradc and lumbar) was defined as the ratio between the number of fused joints and the total number of joints visible in that specific region over all sedions. This proportion was compared between groups for each region, with one to three useable samples per group. Due to the low sample size, no statistical analyses were performed.

### Statistical analyses

Statistical analyses were performed using SPSS (SPSS Statistics 24, IBM corp., Armonk, NY, USA). For the ‘critical timings’ study, to maximise the number of experimental samples that could be obtained, a single pooled control group (named Ctl_E9) was used, with controls undergoing 1 d of PBS treatment at either E3, 4, 5 or 6. No significant differences were present in GC between groups treated with PBS at different days, with most of the GC comparisons being statistically equivalent (data not shown). The numbers of specimens (*n*) harvested and analysed in each group are summarised in Table 2. To assess the critical timings for spine and rib development, GC in each individual vertebra, number of wedged, fused and abnormal vertebrae, rib length and tortuosity were compared between control (Ctl_E9) and immobilised (Im3_E9, Im4_E9, Im5_E9, Im6_E9) groups using one-way ANOVAs followed by Tukey *post-hoc* test, where *p* < 0.05 was considered statistically significant.

For the ‘ontogenetic’ study, the GC in each individual vertebra, spine height, number of wedged, fused and abnormal vertebrae, rib length and rib tortuosity were compared between each immobilised group (Im4_E6, Im4_E7, Im4_E8 and Im4_E9, respectively) and its age-matched control group (Ctl_E6, Ctl_E7, Ctl_E8 and Ctl_E9, respectively) using a two-tailed unpaired *t*-test, where *p* < 0.05 was considered statistically significant.

## Results

### Critical timings of foetal mobility

#### Curvature effects were most severe after immobilisation at E4

**Fig. 3.**
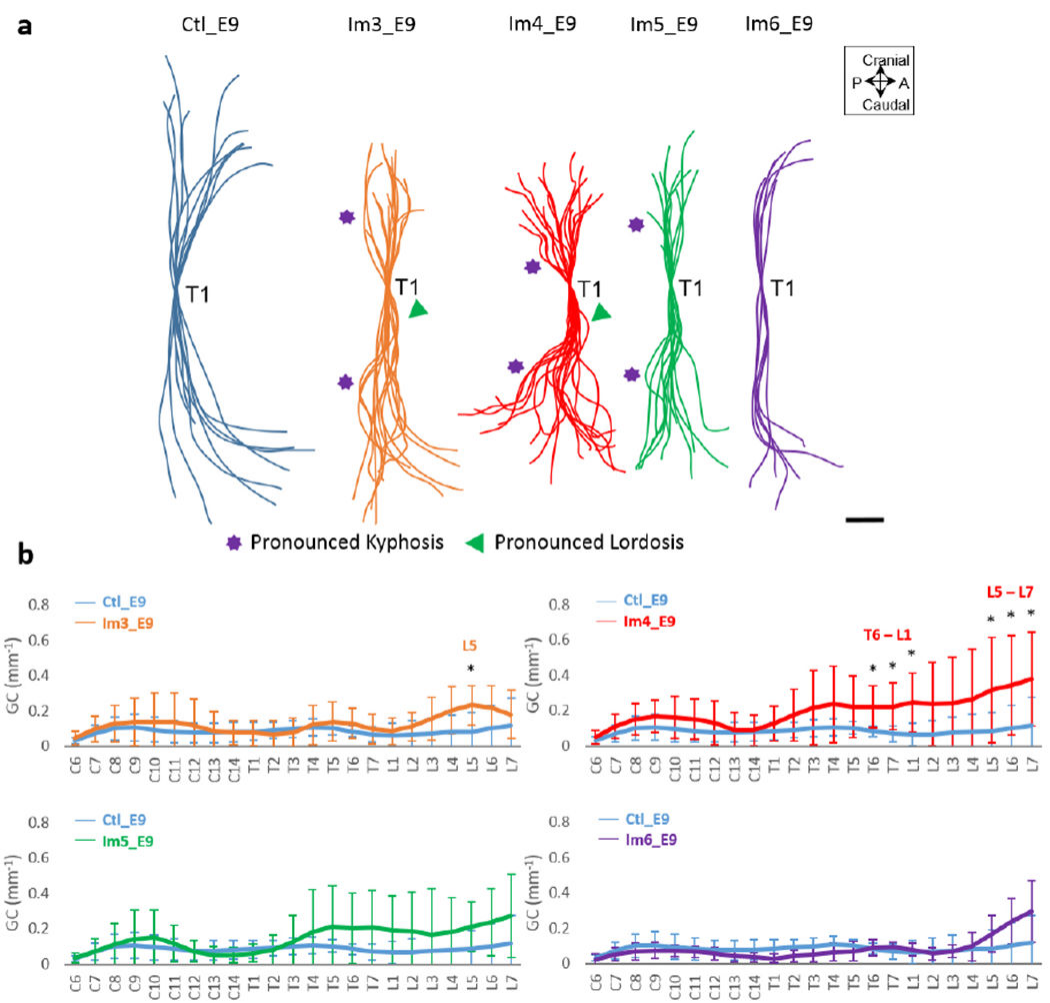
Single-day immobilisation at E4 led to the most severe effects on sagittal spinal curvature. (**a**) Overlays of curvatures in the sagittal plane of control spines (blue), immobilised spines at day 3 (orange), 4 (red), 5 (green) and 6 (purple). Regions of pronounced kyphosis (stars) and lordosis (arrows) are highlighted. Scale bar: 2 mm. (**b**) Absolute GC analysis of control and immobilised spines. Significant differences are identified between single-day immobilisation and control, * *p* < 0.05. A: anterior; P: posterior; C: cervical; T: thoracic; L: lumbar.

Single-day immobilisation at or prior to E5 resulted in a disruption of spinal sagittal curvature (Fig. 3). Immobilisation at E4 [when sclerotome migration is occurring (Christ and Ordahl, 1995)] resulted in multiple regions of pronounced kyphosis and lordosis (Fig. 3a), with significant increases in GC in thoracic and lumbar regions as compared to controls (Fig. 3b). Immobilisation at E3 or E5 resulted in several regions of pronounced kyphosis and/or lordosis (Fig. 3a), but a significant increase in GC was observed only at one location for the E3 regimen, at lumbar vertebra 5 (L5) (Fig. 3b). Immobilisation at E6 did not lead to observable abnormalities in sagittal curvature or any significant differences in GC (Fig. 3a,b).

#### Vertebral anatomy was most severely affected by immobilisation at E3 or E4

**Fig. 4.**
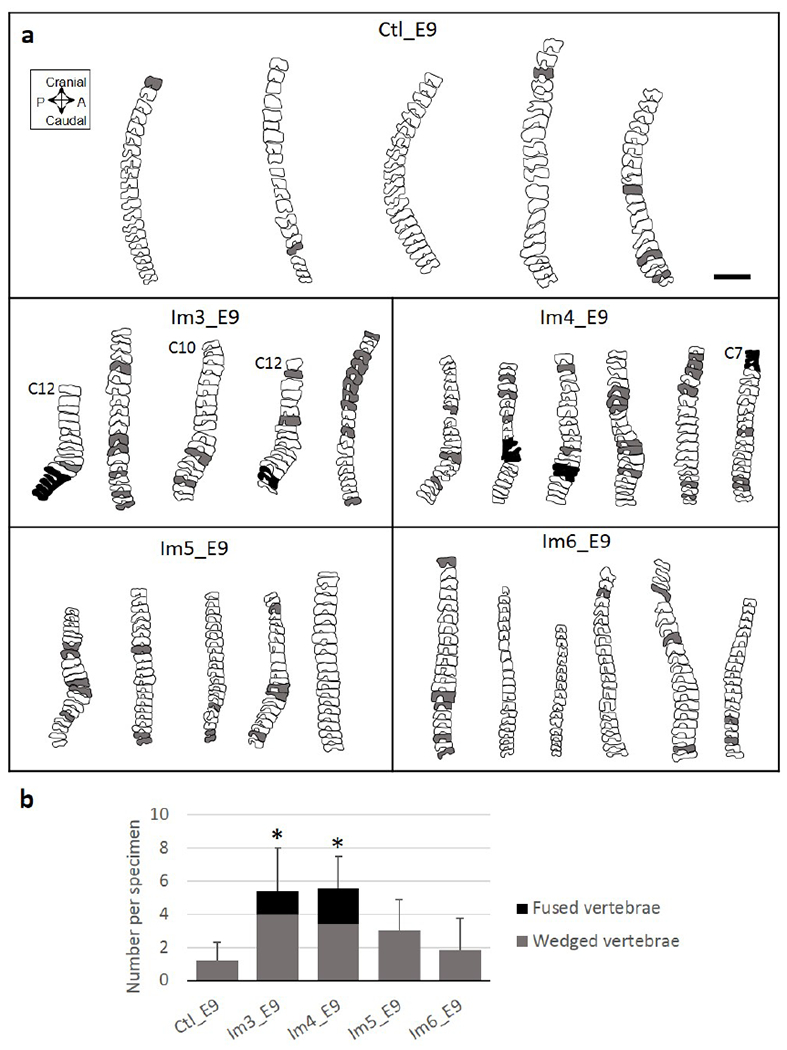
Single-day immobilisation at E3 or E4 induced severe vertebral wedging in the cervical, thoracic and lumbar regions. (**a**) Representative sagittal outlines of the vertebrae from cervical C8 (unless specified) to lumbar L7 of control and single-day-immobilised specimens. Shaded vertebrae indicate a vertebral body angle greater than 10° (relative to distal vertebra, grey) or fused vertebrae (black). Scale bar: 2 mm. (**b**) Bar chart showing mean + standard deviation (SD) numbers of wedged (grey) and fused (black) vertebrae per specimen. Significant differences in total number of abnormal vertebrae between each immobilised group and control group are highlighted with an asterisk, where * *p* < 0.05. A: anterior; P: posterior.

**Fig. 5.**
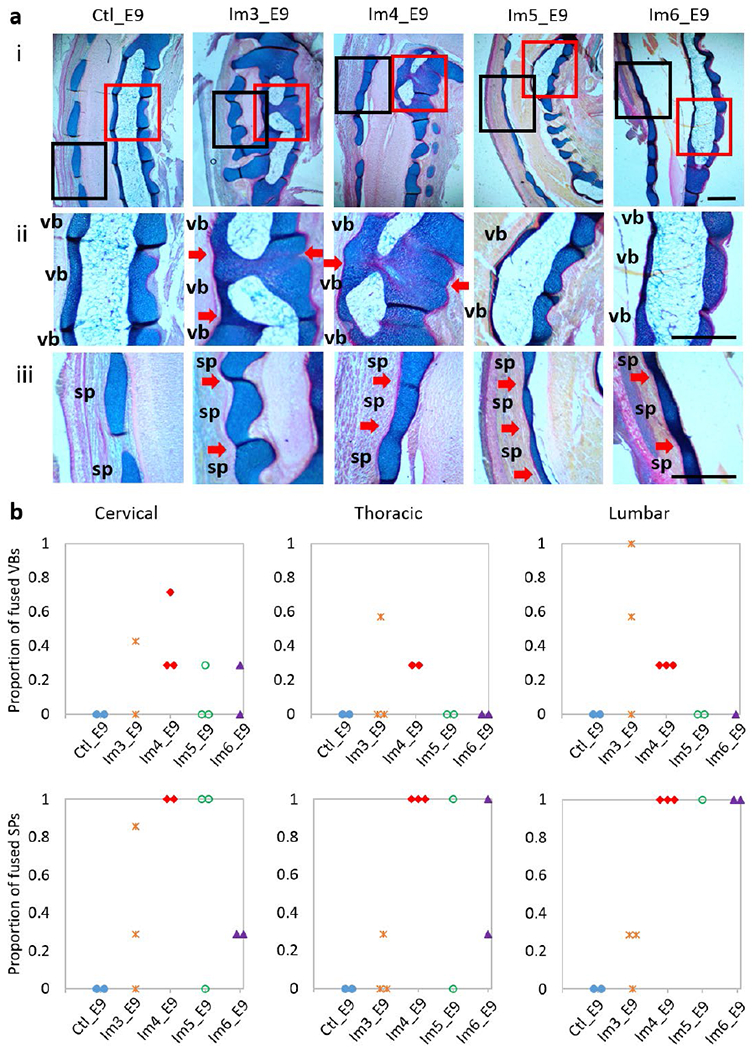
Single-day immobilisation at E3 or E4 induced segmentation defects of vertebral bodies in all regions examined, while single-day immobilisation at any day between E3 and E6 induced fusion of spinous processes. (**a**) Representative sagittal altian blue (cartilage)- and picrosirius red (collagen)-stained sections of a spinal segment (i), vertebral body (ii, red box in i) and spinous process (iii, black box in i) joints in the cervical region of control and single-day-immobilised spines. Red arrows indicate fusion of vertebral bodies or spinous processes, sp: spinous process; vb: vertebral body. Scale bars: 500 μm. (**b**) Dot plots representing the proportion of fused vertebral bodies (VBs) and spinous processes (SPs) of each specimen in the regions examined.

From the 3D data, very few wedged vertebrae and no fused vertebrae were observed in control specimens (Fig. 4a). Single-day immobilisation at E3 and E4 resulted in multiple wedged vertebrae and some fusion of adjacent vertebrae (Fig. 4a). The total number of abnormal vertebrae in both groups was significantly larger than in the control group, with approximately five abnormal vertebrae, on average, per specimen for the Im3_E9 and Im4_E9 groups (Fig. 4b). Specimens immobilised for a single day at E5 and E6 had fewer wedged vertebrae (on average, approximately three per specimen in the Im5_E9 group and two in the Im6_E9 group), but no fusion of adjacent vertebrae (Fig. 4a). No significant differences in the number of abnormal vertebrae between Im5_E9 or Im6_E9 and the control group were observed (Fig. 4b). Histological analyses of the control group revealed normal segmentation of vertebral bodies and spinous processes in all regions examined (cervical, thoracic and lumbar) (Fig. 5a,b). Immobilisation at E3 or E4 caused segmentation defects in the vertebral bodies of all examined regions (Fig. 5b), with complete fusion of some adjacent vertebrae (Fig. 5a). In particular, all specimens immobilised at E4 exhibited some fused vertebral bodies, with the proportion of fused joints ranging from 0.3 to 0.7 in the three regions examined. A few specimens immobilised at E5 or E6 displayed some vertebral body segmentation defects in the cervical region but had normal vertebral body segmentation in the thoracic and lumbar regions (Fig. 5b). These results were consistent with the fusion of vertebral bodies observed in Im3_E9 and Im4_E9 by OPT. None of the immobilised groups exhibited completely normal spinous process segmentation in any of the three regions examined (Fig. 5a,b), while immobilisation at E4 led to complete fusion of spinous processes throughout the examined regions (Fig. 5b). All specimens immobilised between E4 and E6 displayed complete fusion of spinous processes in the lumbar-region (Fig. 5b).

#### Vertebral rib development was most severely affected by immobilisation at E5

**Fig. 6.**
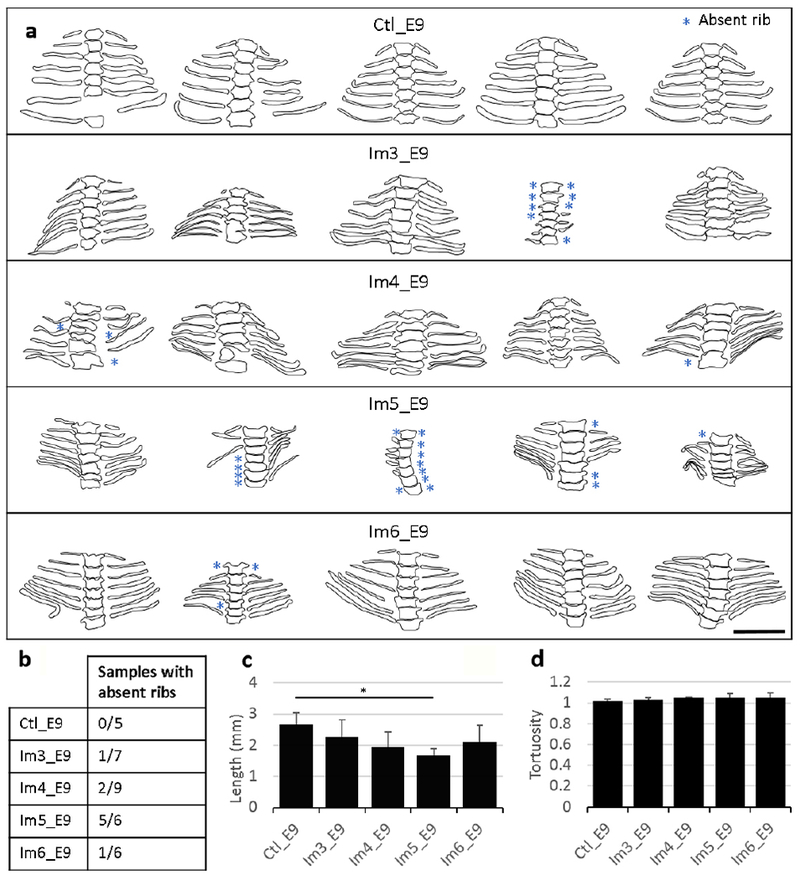
Single-day immobilisation at E5 led to severe abnormalities in vertebral rib development. (**a**) Frontal outlines of the thoracic vertebrae and associated vertebral ribs of control and single-day-immobilised specimens. Blue stars indicate absent ribs. No fused ribs were found. Scale bar: 2 mm. (**b**) Number of samples with at least one absent rib as a proportion of number of samples analysed, (**c**) Bar chart showing length of the fifth left vertebral rib of control and immobilised specimens, (**d**) Bar chart showing mean + SD tortuosity of the fifth left vertebral rib of control and immobilised specimens, (**c**) Significant differences in rib length and (**d**) rib tortuosity between each immobilised group and control group are highlighted with an asterisk, where * *p* < 0.05.

Immobilisation at E5 [when chondrocyte differentiation is occurring in ribs (Winslow and Burke, 2010)] resulted in severe effects on vertebral rib development, with 5 out of 6 specimens showing absent ribs (Fig. 6a,b). 2 out of 9 specimens immobilised at E4 displayed one or several absent ribs, while only 1 specimen from both Im3_E9 (*n* = 7) and Im6_E9 (*n* = 6) displayed absent ribs. The average length of the fifth left vertebral rib decreased significantly in specimens immobilised at E5 as compared to controls, going from 2.7 ± 0.36 mm in control group to 1.7 ± 0.22 mm in Im5_E9 (Fig. 6c). No significant differences in the average vertebral rib tortuosity were measured between specimens immobilised at E3, E4, E5 or E6 as compared to the control group (Fig. 6d). None of the groups displayed rib fusion (data not shown). From the rib outlines, the spacing between some adjacent ribs seemed reduced in specimens immobilised between E3 and E5 (Fig. 6a).

### Ontogenetic effects following foetal immobility at E4

#### Spinal curvature affected by E5 and spinal height decreased by E8

**Fig. 7.**
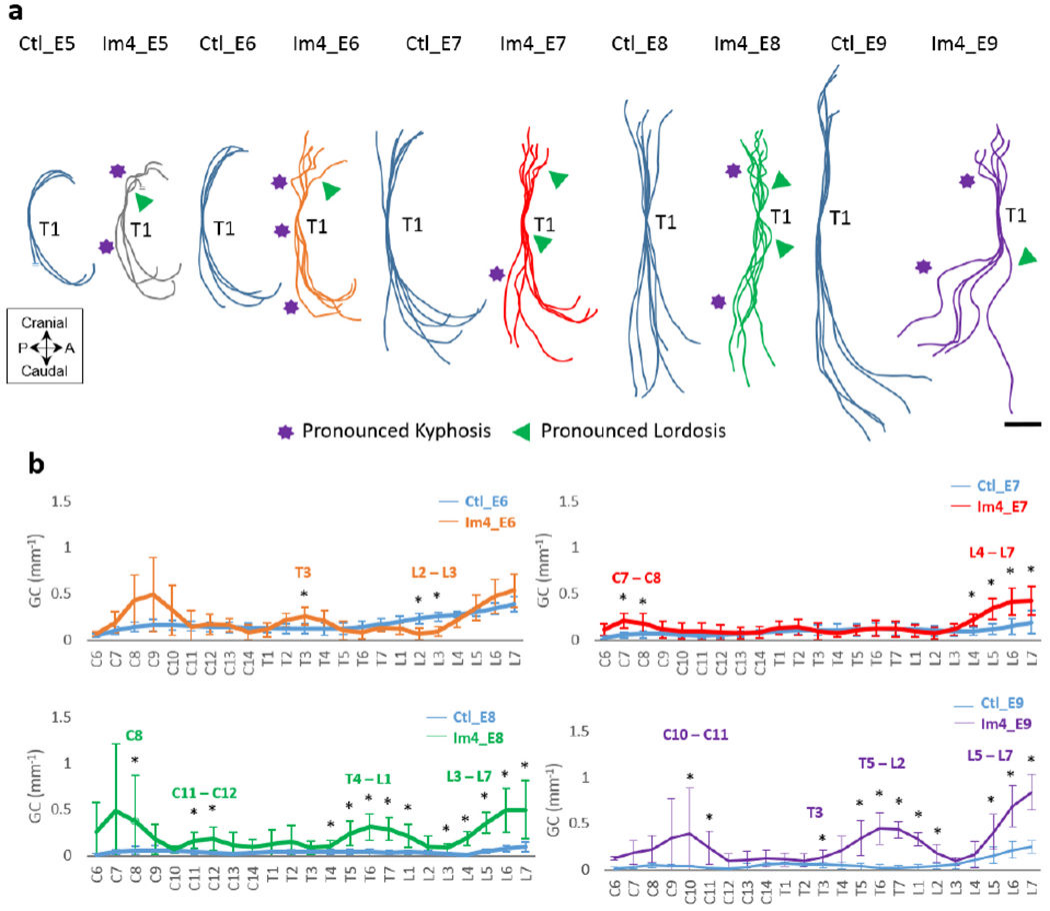
Immobilisation for a single day at E4 had effects on curvature that became progressively more severe as development progressed. (**a**) Overlays of curvatures in the sagittal plane of control spines (blue) and immobilised spines at E4 and harvested at E5 (grey), E6 (orange), E7 (red), E8 (green) and E9 (purple). Regions of pronounced kyphosis (stars) and lordosis (arrows) are highlighted. Scale bar: 2 mm. (**b**) Absolute GC analysis of control and immobilised spines. GC was not quantitatively assessed at E5, due to the lack of vertebral definition at this age. Significant differences identified are between single-day immobilisation and control, * *p* < 0.05. A: anterior; P: posterior; C: cervical; T: thoracic; L: lumbar.

GC was only quantified from E6, due to the lack of vertebral definition at E5. Nonetheless, abnormalities in sagittal curvature were visible at E5 in immobilised specimens, with sub-regions of the cervical region showing pronounced kyphosis and lordosis (Fig. 7a). Curvature abnormalities became progressively more severe until E9, with multiple sub-regions exhibiting pronounced kyphosis and lordosis in cervical, thoracic and lumbar regions. Significant differences in sagittal GC were identified at three vertebral locations, T3 and L2-L3, in the Im4_E6 group and at six vertebral locations in the Im4_E7 group, C7-C8 and L4-L7 (Fig. 7b). In the Im4_E8 and Im4_E9 groups, all regions examined (cervical, thoracic and lumbar) displayed significant changes in curvature, with 13 vertebral locations in the Im4_E8 group and 11 vertebral locations in the Im4_E9 group exhibiting significant differences. In the control group, spinal height increased continuously, from 6.2 ± 0.4 mm at E6 to 10.9 ± 0.6 mm at E9, corresponding to normal development. Spinal height of the immobilised specimens was similar to the control group at E6 and E7, but significantly decreased when compared to controls at E8 and E9 (Fig. 10).

#### Effects on vertebral anatomy visible by E6

**Fig. 8.**
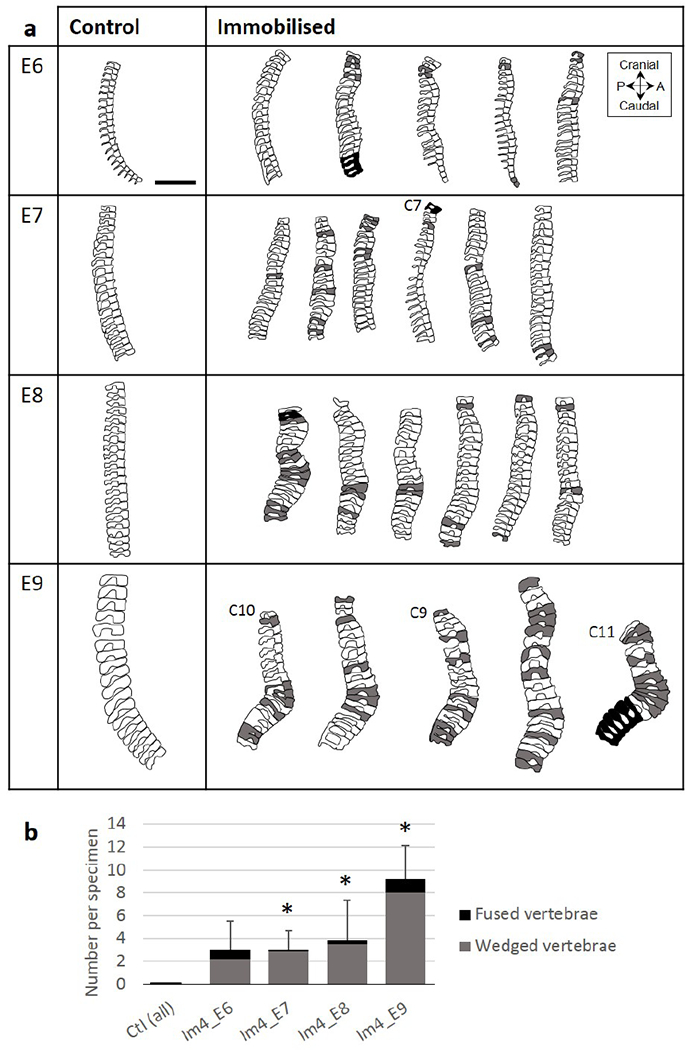
After immobilisation at E4, vertebral wedging was visible by E6 and got progressively more severe until E9. (**a**) Sagittal outlines of the vertebrae from cervical C8 to lumbar L7 (unless specified) of control and immobilised specimens harvested at E6, E7, E8 and E9. Shaded vertebrae indicate a vertebral body angle greater than 10° (relative to distal vertebra, grey) or fused vertebrae (black). Scale bar: 2 mm. (**b**) Bar chart showing mean + SD numbers of wedged (grey) and fused (black) vertebrae per specimen. Significant differences in number of wedged vertebrae between immobilised and age-matched control groups were identified, * *p* < 0.05.

**Fig. 9.**
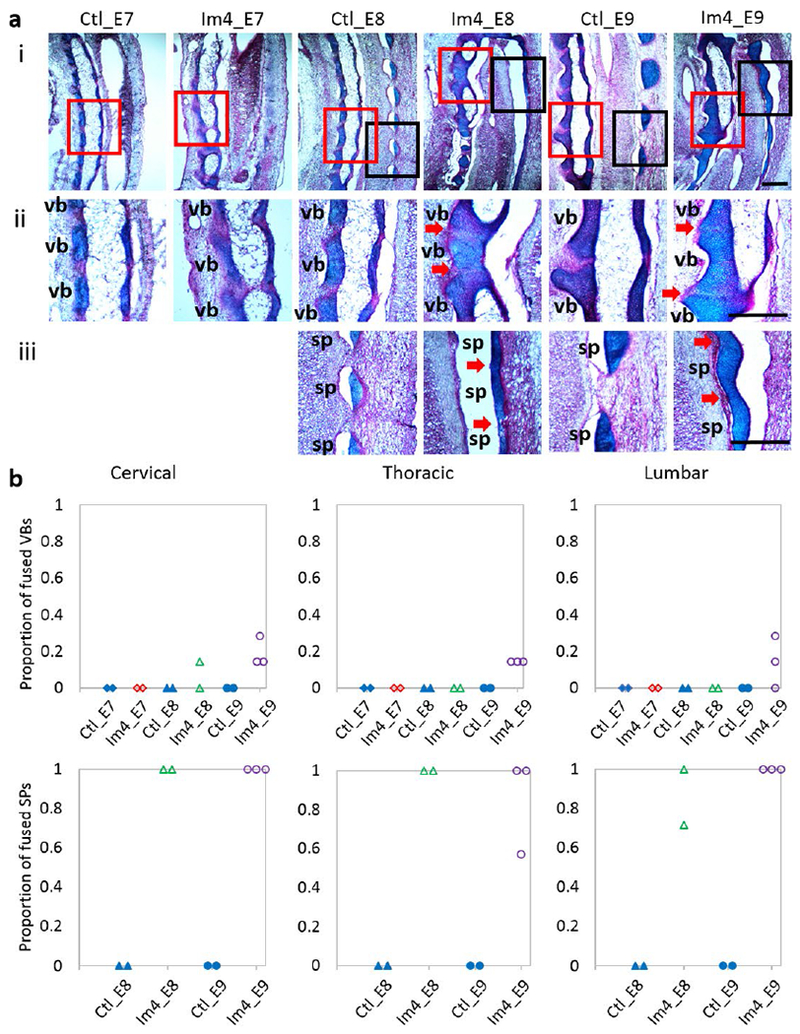
Immobilisation at E4 led to i) fusion of vertebral bodies in the cervical region by E8 and in all regions examined at E9, in a proportion up to 0.3 and ii) fusion of spinous processes in all regions examined as they formed at E8, in a proportion ranging from 0.57 to 1. (**a**) Representative sagittal alcian blue (cartilage)- and picrosirius red (collagen)-stained sections of a spinal segment (i), vertebral body (ii, red box in i) and spinous process (iii, black box in i) in the cervical region of control and single-day-immobilised spines. Spinous processes were visible by E8. Red arrows indicate fusion of vertebral bodies or spinous processes. sp: spinous process; vb: vertebral body. Scale bars: 500 μm. (**b**) Dot plots representing the proportion of fused vertebral bodies (VBs) and spinous processes (SPs) of each specimen in the regions examined.

**Fig. 10.**
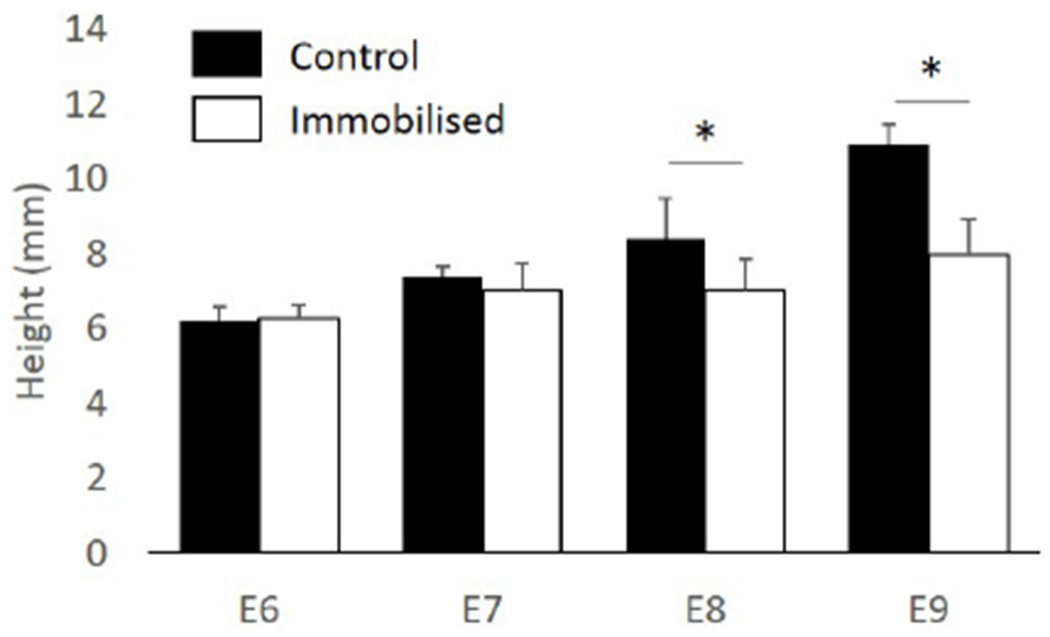
Immobilisation at E4 led to a significant decrease in spinal height from E8. Spinal height measured from cervical vertebra C8 to lumbar vertebra L7 in control and immobilised groups, harvested at E6, E7, E8 and E9. Mean + SD shown, * *p* < 0.05.

The 3D data showed no wedged or fused vertebrae in any of the control specimens across development (Fig. 8). However, in immobilised specimens, vertebral wedging was visible in the cervical region by E6 and in all regions by E7 (Fig. 8a). The average number of wedged vertebrae per specimen was significantly larger in immobilised groups than in controls by E7 and it progressively increased during development, from 2.8 ± 1.8 at E7 to 8 ± 1.2 at E9 (Fig. 8b). One specimen in each experimental group displayed vertebral fusion (Fig. 8a), but the overall number of fused vertebrae in immobilised groups was not significantly different from controls (Fig. 8b). From the histological data, a clearly defined cartilaginous region of the developing vertebral body was observed by E7 in both control and immobilised groups, while spinous processes were observed at E8 (Fig. 9a). Vertebral bodies and spinous processes were fully segmented in all control specimens between E7 and E9 (Fig. 9b). After immobilisation at E4, vertebral body segmentation was initially normal at E7, with fusion becoming increasingly apparent until E9, while spinous process joints were fused as soon as they formed. Immobilised groups displayed normal segmentation of vertebral bodies at E7 in all regions examined (Fig. 9a,b). At E8, incomplete segmentation was observed in the cervical region of only one specimen (Fig. 9a,b), while at E9, incomplete vertebral body segmentation was seen in all three regions examined for all but one specimen (Fig. 9a,b). However, the proportions of fused vertebral bodies were low, not exceeding 0.3 (Fig. 9b). As soon as the spinous processes were visible (at E8), they were observed to be completely fused in the cervical region and partially or completely fused in the thoracic and lumbar regions of the immobilised specimens (Fig. 9a,b).

#### Delayed and abnormal rib formation

**Fig. 11.**
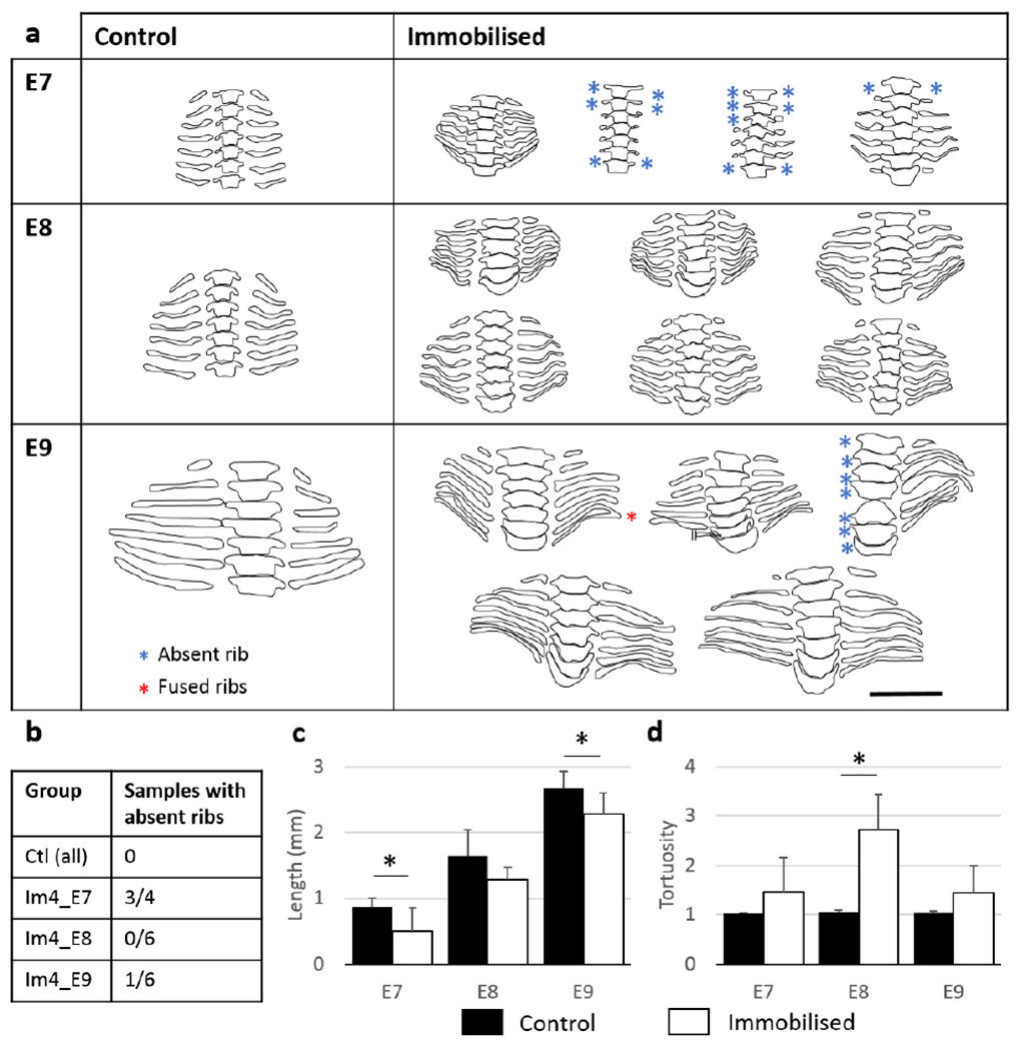
Immobilisation at E4 led to delayed rib formation and abnormal rib tortuosity. Frontal outlines of the thoracic vertebrae and associated vertebral ribs of control and single-day-immobilised specimens harvested at E6, E7, E8 and E9. Absent ribs (blue stars) and fused ribs (red stars) are indicated. Scale bar: 2 mm. (**b**) Number of samples with at least one absent rib as a proportion of number of samples analysed. Bar charts showing (**c**) mean length and (**d**) tortuosity of the fifth left vertebral rib of control and immobilised specimens. SD shown, * *p* < 0.05.

The early stages of cartilaginous rib formation were observed at E6 (data not shown) and all ribs were apparent by E7 in the control group (Fig. 11a,b). Immobilisation at E4 resulted in delayed rib formation, with only one specimen displaying seven cartilaginous ribs on each side at E7 (Fig. 11a,b). Moreover, ribs appeared to be shorter in immobilised specimens at all stages, although differences were significant only at E7 and E9 (Fig. 11c). Immobilisation also resulted in changes in rib shape, especially at E7 and E8. Control specimens showed curved ribs with a single smooth curve, while immobilised specimens showed wavy ribs, with several small curves (Fig. 11a). This resulted in a significant increase in the tortuosity of the fifth left vertebral rib at E8 (Fig. 11d). All ribs were present at E8 and only one specimen displayed absent ribs at E9 (Fig. 11a,b). One immobilised specimen displayed two fused ribs at E9 (Fig. 11a) and the spacing between adjacent ribs seemed reduced in most of the specimens at E8 and E9 (Fig. 11a).

## Discussion

The present study demonstrated for the first time that 24 h of foetal immobility induced between E3 and E5 could have major consequences on spinal curvature, vertebral shape and segmentation and rib development in the chick. The primary hypothesis, that the timing of immobility differentially affected distinct features of spine and rib development, was corroborated. The effects of timing on the various structures examined are summarised in Table 3. Movements at E3 and E4 were most critical for spinal curvature, vertebral shape and segmentation, whilst movements at E5 were most critical for rib development. The second hypothesis, that later aspects of spine and rib development depended on earlier events, was partially corroborated. Results suggested that segmentation of vertebral bodies and spinous processes depended on spinal curvature and vertebral shape, but that rib development was independent from thoracic vertebral anatomy or curvature changes. Altogether, these results highlight the role of foetal mobility across early stages of spine and rib development and the inter-relationships between the various aspects of spine development.

The results of the present study bring new insight into the role of foetal mobility during the early stages of spine development. In chick embryos, movements start at E3.5 (Hamburger and Balaban, 1963). The present study showed that foetal immobility at E4 led to severe effects on spinal curvature immediately after the period of immobilisation and that immobilisation at E3 or E5 led to moderate effects at E9. These results demonstrated that the first foetal movements were critical for spinal curvature. This finding also supports the concept that mechanotransduction and muscle control by the proprioceptive system is required for spinal alignment (Blecher et al., 2017). Immobilisation at E6 did not have any evident effects on spinal curvature. Another possible explanation for the quantified curvature defects is that the reduction of muscle growth induced by rigid paralysis (Macharia et al., 2004) might have physically restrained the growing spine. Foetal movements in the chick start in the neck and extend to the base of the leg buds by E4 (Hamburger and Balaban, 1963) and abnormal curvature at E5 was first observed only in the cervical region. Even though immobilisation was induced for a short time, effects of foetal immobility were not rescued but became progressively more severe and extended to the thoracic and lumbar regions as the spine grew. This study showed lack of recovery so that, once spinal deformity initiates, it is likely to propagate in a vicious cycle (Stokes, 2007).

The critical timings of movements identified correlate with timings of sclerotome cell migration and differentiation in the avian embryo (Christ et al., 2000). The vertebral column develops from the somites, which give rise to the sclerotome between E2 and E3 (Christ et al., 2000; Christ and Ordahl, 1995; Scaal, 2016; Scaal and Christ, 2004). Sclerotome cells migrate dorsomedially from E3 to E4 (Christ and Ordahl, 1995), commencing early cartilage cell differentiation by E5 (Shapiro, 1992), and progressively lead to the formation of vertebral body, rib and spinous process (Scaal, 2016). Immobilisation at E3 or E4 had severe effects on the anatomy of the vertebral bodies, in which cartilage cell differentiation has started by E5 (Shapiro, 1992). Based on this result, it is speculated that dynamic muscle contractions are critical for the migration and differentiation, occurring over E3 (migration) and E4 (differentiation), of the ventral sclerotome forming the vertebral bodies. Moreover, rib development was most strongly affected by the absence of movements at E5. In a chick study, Winslow and Burke (2010) show that Sox9, which is required for chondrocyte differentiation (Akiyama et al., 2002), is expressed in the rib primordia as early as HH25 [Hamburger and Hamilton stage 25 (Hamburger and Hamilton, 1992), corresponding to E4.5/E5] and its expression extends toward the sternum as the development continues. Cartilaginous ribs are visible by E6.5 (Shapiro, 1992), so the results of the present study suggest that movements on E5 are critical for expansion and differentiation of the rib progenitor cells. Finally, immobilisation at E6 affected mainly the spinous processes, which show cartilage definition by E7 (Shapiro, 1992). This result further supports the link between muscle contraction and cell migration and differentiation.

Another important finding of the study was the progression of segmentation defects observed as the spine developed after short-term immobilisation at E4. Segmentation defects are a common feature of congenital abnormalities of the spine and they usually occur when two adjacent somites do not separate correctly (Kaplan et al., 2005). However, the findings of the ontogenetic effects following single-day immobilisation at E4 suggested that fusions of vertebral bodies and spinous processes could occur as a consequence of curvature defects and abnormal vertebral shape, rather than due to abnormal separation of the somites. In normal chick development, individual vertebrae can be seen clearly throughout the length of the spine by E6 (Shapiro, 1992). In the present study, the histological analyses performed at E7 (of specimens immobilised at E4) revealed normal segmentation of vertebral bodies, suggesting that separation of the somites occurred normally after immobilisation. However, some fusion of vertebral bodies was observed at E8 in the cervical region and at E9 in all regions examined (cervical, thoracic and lumbar) of immobilised specimens. Moreover, spinous processes showed complete fusion by E8, as they formed. Based on these results, it is possible that vertebral bodies and spinous processes became fused as cartilage expanded, perhaps due to a lack of space caused by decreased spine height, abnormal shape or spinal curvature defects. Indeed, spine height was significantly decreased by E8 in the specimens immobilised at E4 and the effects of immobilisation on curvature and vertebral shape became severe at E8, when fusion of vertebral bodies and spinous processes was observed.

The relationship between early spine development and rib development was also elucidated. Several studies suggest that rib anomalies and congenital deformities of the spine are closely related (Canavese and Dimeglio, 2013; Dimeglio and Canavese, 2012; Tsirikos and McMaster, 2005). Surprisingly, the present study suggested that the effects of shortterm foetal immobility on the initial stages of rib development were independent of the effects on thoracic vertebral shape or segmentation. Specimens immobilised at E3 displayed multiple wedged vertebrae and moderate defects of segmentation but normal rib development. Specimens immobilised at E5 displayed slight vertebral wedging and normal segmentation but severe rib abnormalities, including missing ribs at E9. The independence between rib and vertebral anatomy found was consistent with a study by Braun et al. (1992), showing completely fused sternum and truncated ribs, but no striking differences in their vertebral shape, in mice lacking the Myf-5 gene. Several studies on the avian skeleton show that differentiation of the ventral and lateral sclerotome, which give rise to vertebral bodies and ribs, respectively, are not regulated by the same signals (Christ et al., 2000; Henderson et al., 1999). Since differentiation of both aspects occurs at different stages, it is possible that the absence of movement at a specific stage would differentially affect these processes. This hypothesis is supported by a study performed in Splotch ((intragenic deletion of the Pax3 gene) mice, which show abnormalities in the expression of lateral markers of the sclerotome, with medial markers apparently unaffected (Henderson et al., 1999). However, although the analyses performed prior to and at E9 suggested independence between rib and spine development, how curvature and vertebral anatomy affect rib development at later stages still remains unknown. Moreover, only the dorsal portions of the ribs could be examined but immobilisation might affect the ventral portion of the ribs as well. Other paediatric rib abnormalities include change of the vertebra-rib angle (Thulbourne and Gillespie, 1976). This feature was not assessed in the present study because sample processing for OPT imaging requires steps (such as clearing) which would potentially affect this angle.

The similarities and differences between the aspects of abnormal spine development found in the present study and the key features of human congenital spine deformities merit further discussion. A direct comparison between the present study findings and the human condition is challenging since analyses were performed at an early foetal stage, whilst most studies on human spine deformities are performed at a neonate or later stage (Ghandhari et al., 2015; Glass et al., 2002; Greggi et al., 2010). Nonetheless, some of the results were consistent with the human literature. Chick specimens immobilised at or prior to E5 exhibited pronounced lordosis and/or kyphosis, which are commonly associated with arthrogryposis in humans, a condition caused by reduced foetal movements and often associated with CS (Greggi et al., 2010). Human CS is commonly associated with hemivertebrae or unilateral vertebral bars (Drummond and Mackenzie, 1978; Fletcher et al., 2010). While some segmentation defects of vertebral bodies were evident in chick specimens immobilised at E3 or E4, wedged vertebrae were more common. It is possible that these wedged vertebrae can lead to segmentation defects or hemivertebrae as the spine grows. Indeed, Greggi et al. (2010) report a case of arthrogryposis where the patient had CS at the age of 1 without any vertebral anomalies but exhibited multiple vertebral fusions at the age of 14. This hypothesis is consistent with the results of the ontogenetic study, which revealed a significant increase in the number of wedged vertebrae by E7, while segmentation defects were not observed before E8. The major differences between the animal model findings and CS in humans were the segmentation defects of the spinous processes. Fusion of the spinous processes was consistently observed in all immobilised groups, whilst no study of human congenital spine deformity reports this abnormality. A possible explanation is that, in the absence of a nucleus pulposus in the chick intervertebral disc (Bruggeman et al., 2012), mobility of the symphysis joint is increased and the thin joints of the spinous processes are more prone to fusion. Finally, human CS is often associated with missing ribs and fused or bifurcated (bifid) ribs (Ghandhari et al., 2015). In the present study, foetal immobilisation at E5 resulted in missing ribs in around 80 % of the specimens, but rib fusion was rare. It is possible that missing ribs were caused by a formation defect and were evident at an early foetal stage, while adjacent ribs might fuse together as they grew. Indeed, the increase in rib tortuosity, along with the irregular spacing between adjacent ribs (not quantified), could lead to rib fusion at a later stage. This theoiy is supported by an arthrogryposis case study, where a patient with marked kyphoscoliosis is diagnosed with fusion of the costo-vertebral joints at the age of 33 (Jones et al., 2008).

The study was not without limitations. Since all analyses were performed on or prior to E9, the effects of foetal immobility after this time-point still remains unknown. For instance, fusion of spinous processes was observed in immobilised specimens at E8 and E9, which might have later effects on spinal curvature or vertebral shape as the spine grew. Moreover, the study focused on the morphological effects due to immobilisation, but other aspects of development would have likely been affected, such as gene expression, bone formation or tissues’ mechanical properties. Indeed, the increase in early rib tortuosity in specimens immobilised at E4 suggested alterations in their mechanical properties following immobilisation. Moreover, studies on immobilised chicks or mice lacking skeletal muscles reveal that, in the limbs’ developing rudiments, a set of genes are differentially regulated due to foetal immobility (Nowlan et al., 2008; Rolfe et al., 2014) and that bone formation is delayed or shows abnormal patterning (Hosseini and Hogg, 1991; Nowlan et al., 2008). These aspects have been poorly investigated for spine development and will be the focus of future studies. Other limitations of the study were the animal model and the nature of immobilisation. Chick embryos are widely used for developmental studies because the basic mechanisms of the vertebral column development are similar to humans (Christ et al., 2000). However, the chick model is not suitable for investigations on the intervertebral disc, since involution of the notochord does not take place in chickens and their discs lack the nucleus pulposus (Bruggeman et al., 2012). Future studies will be conducted on a murine model to uncover the effects of the absence or reduction of movements on intervertebral disc development. Finally, the results demonstrated variability in effects between the two experimental Im4_E9 groups, which underwent the same treatment. In particular, more severe effects on spinal curvature and vertebral shape were observed in the ontogenetic study. As the experiments were performed at different times of the year and as the external temperature has an influence on chick development (Hamburger and Balaban, 1963), it is possible that seasonal effects, along with increased variability in the ontogenetic study due to the smaller sample size, are responsible for some quantitative differences between the two Im4_E9 groups. Nonetheless, both experiments highlighted the same key effects of immobilisation at E4 on spinal curvature, vertebral anatomy and rib development.

In conclusion, the study highlighted the critical timings of foetal mobility for spinal curvature, vertebral anatomy and rib development. A day-long period of immobilisation in the chick had severe effects on spine and rib development and these effects were not rescued by later movements. In particular, movements were the most critical for spine and rib development in the chick from E4 to E5, the period when cartilage differentiation begins and vertebral definition occurs (Shapiro, 1992). The equivalent critical period in human is likely to be between weeks 6, when vertebral chondrification begins (Muller et al., 1986), and 10, when cartilaginous vertebrae are separated by a rudimentary annulus fibrosus (as reviewed by Tworney and Furniss, 1978). Enhanced monitoring of foetal movements at the early stages of spine and rib development could improve the understanding and prenatal diagnosis of CS and other congenital spine deformities.

## Figures and Tables

**Table 1.** Immobilisation regimen applied. Immobilisation was induced by DMB administration, while controls were administered PBS at the embryonic day (E) of treatment. To determine the critical timings of foetal mobility, embryos were treated once at E3, E4, E5 or E6 and harvested at E9. To assess the ontogenetic effects of foetal immobility, embryos were treated at E4 and harvested daily between E5 and E9. T: treatment. H: harvest.

Study	Group	E0	E1	E2	E3	E4	E5	E6	E7	E8	E9
Critical timings of foetal mobility	Im3_E9				T						H
	Im4_E9					T					H
	Im5_E9						T				H
	Im6_E9							T			H
Ontogenetic effects of foetal immobility	Im4_E5					T	H				
	Im4_E6					T		H			
	Im4_E7					T			H		
	Im4_E8					T				H	
	Im4_E9					T					H

**Table 2.** Numbers of immobilised and control chick embryos harvested and used for each analysis. Only curvature analysis could be performed on samples harvested at E5 (Ctl_E5 and Im4_E5) due to the lack of vertebral definition at this stage. Rib and histological analyses were performed on samples harvested at or after E7, as ribs and visible cartilage were not consistently present prior to this point. In instances where samples were lost or adversely affected by pre-processing, staining or imaging, the total number of specimens listed below in column 2 is greater than the combined numbers for OPT and histological analyses.

Group	Total specimens	OPT analyses			Histology
		Curvature	Wedging	Ribs	Segmentation
**Critical timings of foetal mobility: single-day immobilisation at E3, E4, E5 or E6**					
**Ctl_E9**	13	10	5	5	2
**Im3_E9**	16	11	5	7	3
**Im4_E9**	21	17	7	9	3
**Im5_E9**	17	11	5	6	3
**Im6_E9**	10	6	6	6	4
**Ontogenetic effects: single-day immobilisation at E4**					
**Ctl_E5**	6	3	-	-	-
**Im4_E5**	6	3	-	-	-
**Ctl_E6**	6	4	4	-	-
**Im4_E6**	6	5	5	-	-
**Ctl_E7**	8	6	6	5	2
**Im4_E7**	8	6	6	5	2
**Ctl_E8**	8	6	6	6	2
**Im4_E8**	9	6	6	6	2
**Ctl_E9**	7	5	1	5	2
**Im4_E9**	11	5	5	5	3

**Table 3.** Summary table illustrating the severity of the effects of day-long periods of immobility between E3 and E6 on the features of spine and rib development. 0: none, +: mild, ++: moderate, +++: severe. VB: vertebral body, SP: spinous process.

	Sagittal curvature	Vertebral shape	Segmentation		Rib development
			VB	SP	
**Im3_E9**	++	+++	++	++	+
**Im4_E9**	+++	+++	++	+++	++
**Im5_E9**	++	++	+	+++	+++
**Im6_E9**	0	+	+	+++	+

## Discussion with Reviewer

**Reviewer:** In the discussion of the study limitations, the authors touch on an important difference between chick spine development as compared to other vertebrates and specifically the fact that in chicks the notochord persists as a rod-like structure and does not transform in to the nucleus pulposus. Can the authors speculate a bit further on how immobility during the process of notochordal involution might affect intervertebral disc formation? Additionally, could the authors speculate on how the morphological abnormalities observed might adversely affect the neural elements of the spine?

**Authors:** Vertebral shape and notochord involution are closely related, but the exact process remains unclear (Chan et al., 2014, additional reference). Azsodi et al. (1998, additional reference) propose that the developing vertebral body creates a biomechanical force that pushes the notochord cells into the spaces between the vertebrae. In the absence of collagen type II, which provides the tensile force, the rod-like notochord continues to be present throughout development, resulting in the absence or reduction of the nucleus pulposus. It is possible that abnormal vertebral shape caused by foetal immobility could lead to an abnormal internal swelling pressure, resulting in an asymmetrical or reduced nucleus pulposus.

The severe spinal curvature defects observed in the study, along with vertebral wedging, could lead to a disruption of mechanical stress on the spinal cord. This abnormal loading on the spinal cord could affect the neural elements. In a study on 5-week-old Japanese small game fowl, Shimizu et al. (2005, additional reference) show that kyphotic deformities cause spinal cord compression, especially at the ventral side of the spinal cord, due to continuous mechanical stress caused by the kyphotic deformity, resulting in neuronal loss and demyelination.
